# Recent advancements in the understanding of tetraspanin functions

**DOI:** 10.1007/s00430-020-00687-x

**Published:** 2020-07-23

**Authors:** Luise Florin, Charlotte M. de Winde

**Affiliations:** 1 Institute for Virology and Research Center for Immunotherapy (FZI), University Medical Center of the Johannes Gutenberg-University Mainz, Obere Zahlbacher Strasse 67, 55131 Mainz, Germany; 2 Stromal Immunology Group, MRC Laboratory for Molecular Cell Biology, University College London, Gower Street, London, WC1E 6BT UK

The spatiotemporal coordination of transmembrane proteins plays an important role in an exceptionally wide range of cellular activities, including in all steps of pathogen infection as well as immunological processes. Tetraspanin proteins are the master organizers of membrane proteins, and are therefore involved in physiological and pathophysiological processes, as presented in this special issue “Tetraspanins in Infection and Immunity”. These transmembrane proteins span the membrane four times and form extended subdomains by means of their strong tendency to associate laterally with one another and with different classes of proteins, such as cell surface receptors, immunoglobulins, adhesion and signalling molecules, and proteases [1]. As such, they anchor specific proteins to one site on the cell membrane forming microclusters that then further organize into larger assemblies, the so-called tetraspanin-enriched microdomains. These microdomains enable membrane dynamics, like endocytosis, recycling, exocytosis, cell motility, fusion and signalling. The role of tetraspanins in cell fusion, for example, is described in mammalian reproductive processes and development [2].

Because of their involvement in a wide range of cellular processes, tetraspanins are exploited by many pathogens such as viruses and bacteria during their entry and egress. In addition, dysregulation of normal tetraspanin function leads to diseases like cancer, diabetes, Alzheimer's and autoimmune reactions [3, 4]. This, coupled with their easy accessibility as membrane proteins, means that tetraspanins have a huge potential to serve as therapeutic targets for the development of new treatments in cancer, hematological malignancies and infectious diseases [5–8].

In recent years, it became apparent that tetraspanins define entry sites of hepatitis C virus (HCV), human papilloma virus (HPV), coronavirus, influenza A virus, and human immunodeficiency virus by organizing receptors and other components into viral entry platforms [9, 10]. In this issue, five research studies deepen our understanding of the role of tetraspanins in bacterial and viral infections. Super-resolution and confocal imaging analyses suggest that contact of HPV16 particles with the cell surface triggers the formation of large three-dimensional tetraspanin architectures that contain at least two different tetraspanins, CD151 and CD63, and are connected to filamentous actin [11]. In the same virus and cell system, additional tetraspanins like CD9 not necessarily support infection, but act as negative regulators of the invasion process [12]. In this study, comparative analyses using CD9 low- or high-expressing cells suggest that a specific tetraspanin expression optimum promotes the entry process of the pathogen. The inhibitory role of CD9 and CD81 during pathogen infection is also shown by Elgawidi and colleagues [13]. *Burkholderia thailandensis* is able to induce the formation of multinucleated giant cells where these tetrapanins are involved. The authors used tetraspanin antibodies and recombinant proteins corresponding to the large extracellular domain of the tetraspanins to modulate their function. They show that antibodies against CD9 and CD81 enhanced the cell–cell fusion process induced by the bacterium whereas recombinant tetraspanin proteins acted in an inhibitory way.

In addition to their function in plasma membrane processes, tetraspanins also regulate intracellular processes by the modulation of signalling pathways [1]. Benayas and colleagues show that the loss of CD81 on herpes simplex virus type-1 infected cells compromised replication of viral DNA and formation of new infectious particles [14]. The relevance of tetraspanins in naturally occurring infections and their outcome is additionally supported by the study of Alberione et al. [15]. They provide evidence that genetic host-variation contributes to inter-individual differences in HCV infection and outcome.

Tetraspanins are involved in many aspects of immunity, and as such play a pivotal role in establishing an effective immune response [16–18]. Further overview articles in this special issue highlight that tetraspanins control different stages of the migration of dendritic cells, which engulf and present antigens to initiate an immune response, from the site of infection to the lymph node [19]. On mast cells, important in antiviral responses and hyperactive in patients with allergies, tetraspanins control release of intracellular granules with immunomodulatory compounds by membrane remodelling [20]. Two tetraspanins, CD37 and CD53, are exclusively expressed on immune cells [21]. The role of CD37 has been studied extensively in the past two decades, but studies investigating the function of CD53 have only emerged in recent years. In this issue, Dunlock has provided a detailed review on the multifunctional role of CD53 in the immune system, controlling immune cell adhesion and migration, and intracellular signalling pathways [22].

New functional roles for tetraspanins are continuously being discovered, and possibilities for targeting tetraspanins in diseases are emerging. McLaughlin and colleagues review an important role for Tspan7 in the autoimmune response in type 1 diabetes, and propose targeting Tspan7 as a promising strategy to prevent disease [4]. Furthermore, Gavin and colleagues present in this issue Tspan18 as a new regulator of calcium signalling in activated endothelial cells, thereby controlling thrombo-inflammation in acute organ damage upon ischaemic stroke and venous thrombosis [23]. Targeting Tspan18 may be a better potential therapeutic strategy to interfere with endothelial function than targeting Orai1 which is widely expressed on a diverse range of cell types.

Tetraspanin biology is intensively studied since the discovery of this protein family in the mid 1980s, and has developed into a hot topic with several interfaces between structural molecular biology and a variety of diseases. New imaging technologies, such as superresolution microscopy or simulations of molecular dynamics, in combination with functional analysis now enable scientists to understand key mechanisms in the formation of tetraspanin-enriched microdomains and tetraspanin-regulated processes. This will significantly contribute to the discovery of promising tetraspanin targets to treat infections, immunological pathologies, and other diseases. The aim of this special issue is to update the reader in the latest findings concerning the function of tetraspanins in different physiological and pathological situations, focussing on infection and immunity.

Luise Florin

Charlotte M. de Winde

MMIM Guest Editors
